# Salt secretion is linked to acid-base regulation of ionocytes in seawater-acclimated medaka: new insights into the salt-secreting mechanism

**DOI:** 10.1038/srep31433

**Published:** 2016-08-11

**Authors:** Sian-Tai Liu, Jiun-Lin Horng, Po-Yen Chen, Pung-Pung Hwang, Li-Yih Lin

**Affiliations:** 1 Department of Life Science, National Taiwan Normal University, Taipei, Taiwan; 2 Department of Anatomy and Cell Biology, Taipei Medical University, Taipei, Taiwan; 3 Institute of Cellular and Organismic Biology, Academia Sinica, Taipei, Taiwan

## Abstract

Ionocytes in the skin and gills of seawater (SW) teleosts are responsible for both salt and acid secretion. However, the mechanism through which ionocytes secrete acid is still unclear. Here, we hypothesized that apical Na^+^/H^+^ exchangers (NHE2/3), carbonic anhydrase (CA2-like), and basolateral HCO_3_^−^/Cl^−^ exchanger (AE1) are involved in acid secretion. In addition, the hypothesized involvement of basolateral AE1 suggested that acid secretion may be linked to Cl^−^ secretion by ionocytes. The scanning ion-selective electrode technique (SIET) was used to measure H^+^ and Cl^−^ secretion by ionocytes in the skin of medaka larvae acclimated to SW. Treatment with inhibitors of NHE, CA, and AE suppressed both H^+^ and Cl^−^ secretion by ionocytes. Short-term exposure to hypercapnic SW stimulated both H^+^ and Cl^−^ secretion. mRNA of CA2-like and AE1 were localized to ionocytes in the skin. Branchial mRNA levels of NKCC1a, CA2-like, and AE1a increased together with the salinity to which fish were acclimated. In addition, both AE1a and AE1b mRNA increased in fish acclimated to acidified (pH 7) SW; NKCC1a mRNA increased in fish acclimated to pH 9 SW. This study reveals the mechanism of H^+^ secretion by ionocytes, and refines our understanding of the well-established mechanism of Cl^−^ secretion by ionocytes of SW fish.

Osmoregulation is an essential prerequisite for maintaining body fluid homeostasis in vertebrates. Hypertonic seawater poses significant challenges for osmoregulation in aquatic vertebrates. For successful habitation, osmoregulatory tissues and organs have evolved in seawater vertebrates in response to osmotic challenges. Salt-secreting glands are specialized organs for salt secretion in marine vertebrates, including birds, reptiles, and elasmobranches^1^,^2^. In marine and euryhaline teleosts, the gill is the primary organ of salt secretion^3^.

The physiological mechanism of salt secretion was explicitly studied in the gills of seawater-acclimated fish. Within the branchial epithelia, a population of ionocytes (also called mitochondrion-rich cells or chloride cells) has been identified as the cells responsible for secreting internal Na^+^ and Cl^−^ into the ambient seawater (SW)^3^,^4^,^5^,^6^. The current model for NaCl secretion by ionocytes includes three critical transporters: Na^+^-K^+^-ATPase (NKA) and Na^+^-K^+^-2Cl^−^ cotransporter 1 (NKCC1) in the basolateral membrane and the cystic fibrosis transmembrane conductance regulator (CFTR) Cl^−^ channel in the apical membrane. Basolateral NKCC1 carries 1Na^+^, 1K^+^, and 2Cl^−^ into the cell down the electrochemical gradient provided by the action of NKA. The accumulated intracellular Cl^−^ then exits the cell through the apical CFTR channel, and the interstitial Na^+^ is pushed out of the epithelia through the paracellular pathway by the transepithelial potential^5^,^7^. The current model has been established for several decades and has never been challenged.

In addition to salt secretion, the fish gill is also an important organ for acid-base regulation through transporting acid-base equivalents (H^+^, NH_4_^+^, and HCO_3_^−^) into ambient water^3^,^5^,^7^. In SW fish, a considerable amount of base (HCO_3_^−^) is secreted by the intestine^8^, and the compensatory acid (H^+^) secretion is generally believed to occur in gills^3^,^5^,^9^. In recent studies, several transporters and enzymes, including H^+^-ATPase (HA), Na^+^/H^+^ exchanger (NHE), carbonic anhydrases (CA), and anion exchanger (AE), were found to be involved in the acid secretion mechanism of ionocytes in freshwater (FW) fish^3^,^5^,^7^. However, the mechanism of acid secretion in SW fish is not as clear as that in FW fish.

In SW fish, NHE in the apical membrane of ionocytes is thought to be the major transporter for acid secretion^3^,^5^,^9^. In marine sculpin, NHE2 is localized to the apical membrane of ionocytes^10^. In mangrove killifish (*Kryptolebias marmoratus*)^11^ and Japanese eel^12^, NHE3 is present in the apical membrane of ionocytes. Recently, both NHE2 and NHE3 were identified in ionocytes of SW-acclimated medaka, in which they play a critical role in acid secretion^13^.

CA is an enzyme that catalyzes a reversible reaction of CO_2_ and H_2_O (CO_2_ + H_2_O ↔ HCO_3_^−^ + H^+^); this reaction is critical for acid-base regulation in fish^14^. In zebrafish, two types of CA (CA2a and CA15a) were identified in acid-secreting ionocytes^15^. In SW, CA2-like was identified in branchial ionocytes of flounder (*Platichthys flesus*)^16^, mudskipper (*Periophthalmodon schlosseri*)^17^, and pufferfish (*Tetraodon nigroviridis*)^18^.

In FW fish, a putative AE at the apical side of ionocytes has long been speculated to absorb Cl^−^ and secrete HCO_3_^−3^. However, a dominant AE (AE1b) was identified in the basolateral membrane of acid-secreting ionocytes (HR cells) in zebrafish, suggesting that it may be involved in acid secretion instead of Cl^−^ uptake^19^. In pufferfish (*Tetraodon nigroviridis*) and milkfish (*Chanos chanos*), AE1 is localized to the basolateral membrane of both FW- and SW-type ionocytes^18^,^20^. However, the physiological role of AE1 in the ionocytes of SW fish has not been fully investigated.

The above findings relating to NHE, CA, and AE led to the proposal of a hypothetical mechanism behind acid secretion by SW-type ionocytes: CA2-like promotes the formation of H^+^ and bicarbonate, and then H^+^ is secreted into SW via apical NHE2/3, and bicarbonate is taken back into circulation via basolateral AE1. Although NHE2/3 plays a direct role in transporting H^+^ out of ionocytes, CA2-like and AE1 are required for the complete process, i.e., acid (H^+^) secretion and base (bicarbonate) reclamation. The first aim of the current study was to test this hypothesis. Moreover, ionocytes in SW fish are responsible for both acid-base regulation and salt secretion^13^,^21^,^22^, and therefore Cl^−^ secretion and H^+^ secretion are presumed to occur in the same ionocytes. We thus hypothesized that Cl^−^ is not simply transported by NKCC1, but also by AE1, into ionocytes. In other words, basolateral AE1 plays a dual role in acid-base regulation and salt secretion of ionocytes in SW fish. The second aim of this study was to test this hypothesis.

SW-acclimated medaka larvae were used to test the above hypotheses, because our previous studies established functional approaches for examining ionocytes in the skin of intact larvae^13^,^21^,^23^. A non-invasive electrode technique, the scanning ion-selective electrode technique (SIET), was applied to measure H^+^ and Cl^−^ secretion by the ionocytes in medaka larvae. *In situ* hybridization and immunohistochemistry were used to localize CA2-like and AE1 in ionocytes. Real-time quantitative PCR was applied to analyze the mRNA expression of associated genes in fish gill.

## Results

### Effects of inhibitors (DIDS, AZ, EIPA, and bumetanide) on H^+^ and Cl^−^ secretion of ionocytes

**Figure 1:**
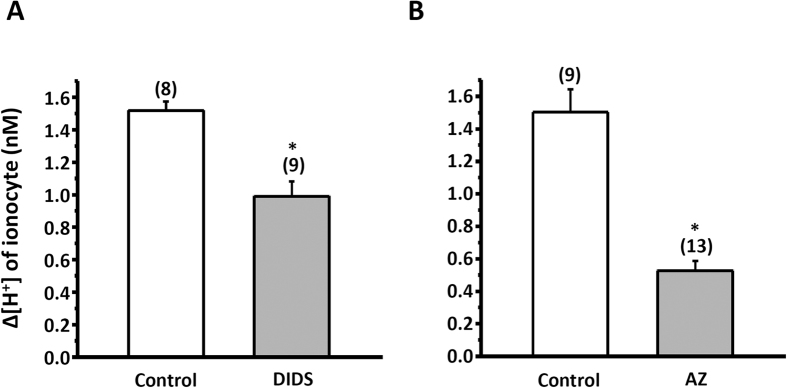
Effects of DIDS (400 μM) (**A**) or AZ (200 μM) (**B**) treatment on Δ[H^+^] at ionocytes of larval skin. Data are presented as the means ± SEM. *p < 0.05 (two-tailed unpaired Student’s *t*-test). The numbers of cells are shown in parentheses, the analyzed cells were sampled from 5–6 larvae.

**Figure 2:**
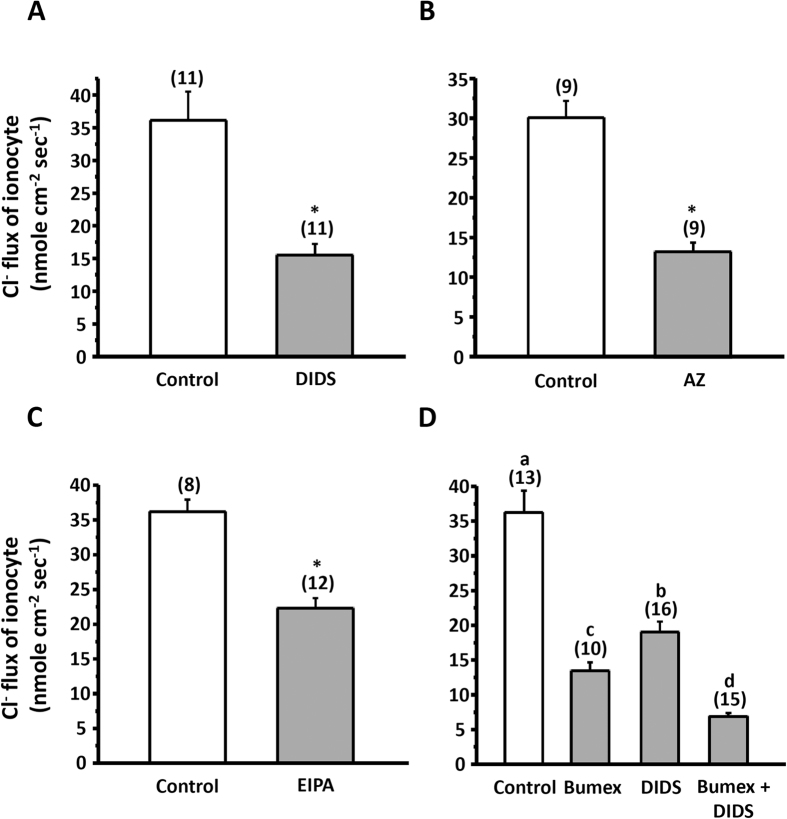
Effects of DIDS (400 μM) (**A**), AZ (200 μM) (**B**), EIPA (400 μM) (**C**), and bumetanide (Bumex; 400 μM) and/or DIDS (400 μM) (**D**) on Cl^−^ flux at ionocytes of larval skin. Data are presented as the means ± SEM. *p < 0.05 (two-tailed unpaired Student’s *t*-test). Different letters indicate a significant difference (one-way ANOVA, Tukey’s comparison, *p* < 0.05). The numbers of cells are shown in parentheses, the analyzed cells were sampled from 5–7 larvae.

The H^+^ gradient (∆[H^+^]; Fig. 1) and Cl^−^ flux (Fig. 2) were measured with SIET at the apical surface of ionocytes in SW-acclimated larvae pretreated with inhibitors (DIDS, AZ, EIPA, or bumetanide) for 30 min. In preliminary experiments, different doses of inhibitors were applied to the larvae to determine the maximal effect on H^+^ and Cl^−^ secretion by the larval skin, and the maximal dosages were used in the following experiments. In this study, we found that DIDS (400 μM) and AZ (200 μM) suppressed 35% and 66% of H^+^ secretion by ionocytes, respectively (Fig. 1A,B). Our previous report revealed that EIPA (200 μM) effectively suppressed 49% of H^+^ secretion by larval skin^13^. These results suggest that NHE, AE, and CA are involved in the acid secretion of ionocytes in SW-medaka. Interestingly, we also observed that Cl^−^ secretion (positive values of Cl^−^ flux) by ionocytes was suppressed by EIPA (38%), DIDS (57%), or AZ (56%) exposure for 30 min (Fig. 2), suggesting that acid secretion is linked to Cl^−^ secretion. To compare the contribution of NKCC and AE to Cl^−^ secretion by ionocytes, we treated larvae with bumetanide (400 μM) and/or DIDS (400 μM); bumetanide and DIDS suppressed Cl^−^ secretion by 63% and 47%, respectively, while simultaneous treatment with both inhibitors suppressed it by up to 81% (Fig. 2D).

### Effects of CO_2_ exposure on H^+^ and Cl^−^ secretion of ionocytes

**Figure 3:**
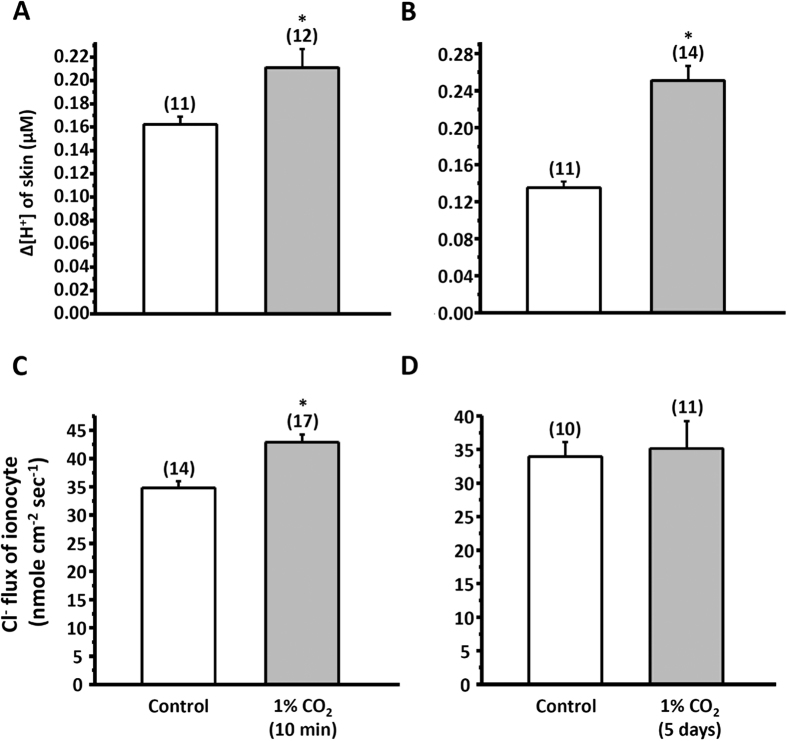
Effects of 1% CO_2_ treatment on ∆[H^+^] (**A,B**) and Cl^−^ flux (**C,D**) at ionocytes of larval skin after short-term (10 min) or long-term (5 days) exposure. Data are presented as the means ± SEM. *p < 0.05 (two-tailed unpaired Student’s *t*-test). The numbers of cells are shown in parentheses, the analyzed cells were sampled from 6–9 larvae.

To further demonstrate the association between H^+^ and Cl^−^ secretion, we exposed medaka larvae to hypercapnia SW for a short time (10 min), which was expected to stimulate H^+^ secretion by ionocytes. Indeed, incubation in 1% CO_2_ resulted in a significant increase in H^+^ secretion by larval skin (Fig. 3A) and Cl^−^ secretion by ionocytes (Fig. 3C). However, an association between H^+^ and Cl^−^ secretion was not observed in larvae acclimated to 1% CO_2_ for 5 d: H^+^ secretion was significantly enhanced, but Cl^−^ secretion was unaffected by such long-term incubation (Fig. 3B,D).

### Localization of AE1 and CA2-like in the yolk sac skin of larvae.

**Figure 4:**
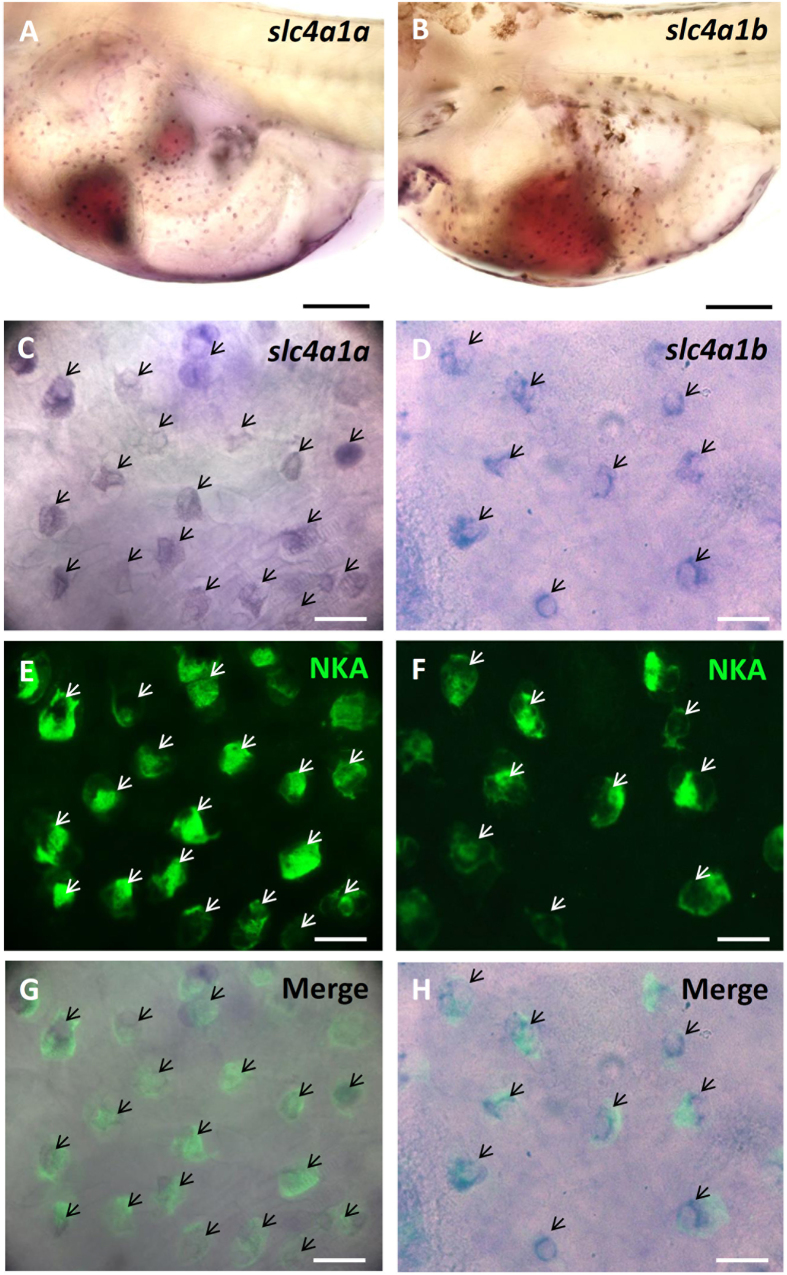
Localization of *slc4a1a* and *slc4a1b* mRNA in ionocytes of larval skin. Whole-mounted *in situ* hybridization was used to visualize the mRNA of *slc4a1a* (**A,C,G**) and *slc4a1b* (**B,D,H**). Na^+^/K^+^-ATPase (NKA) immunocytochemistry (**E,F**) was used to label ionocytes. The merged images indicate that *slc4a1a* and *slc4a1b* were co-expressed with NKA (arrows). Scale bar: 10 μm (**C**–**F**) and 100 μm (**A,B**).

Two *slc4a1* (AE1) paralogs (*slc4a1a* and *slc4a1b*) were found in the medaka genome. *In situ* hybridization was used to localize the *slc4a1a* (AE1a) and *slc4a1b* (AE1b) transcripts in SW-acclimated larvae. We observed that both *slc4a1a* (Fig. 4A,C,G) and *slc4a1b* (Fig. 4B,D,H) transcripts were present in dispersed ionocytes which expressed NKA (Fig. 4E,F). Negative controls with sense probes did not show signals in the larvae (supplementary Fig. S1.)

**Figure 5:**
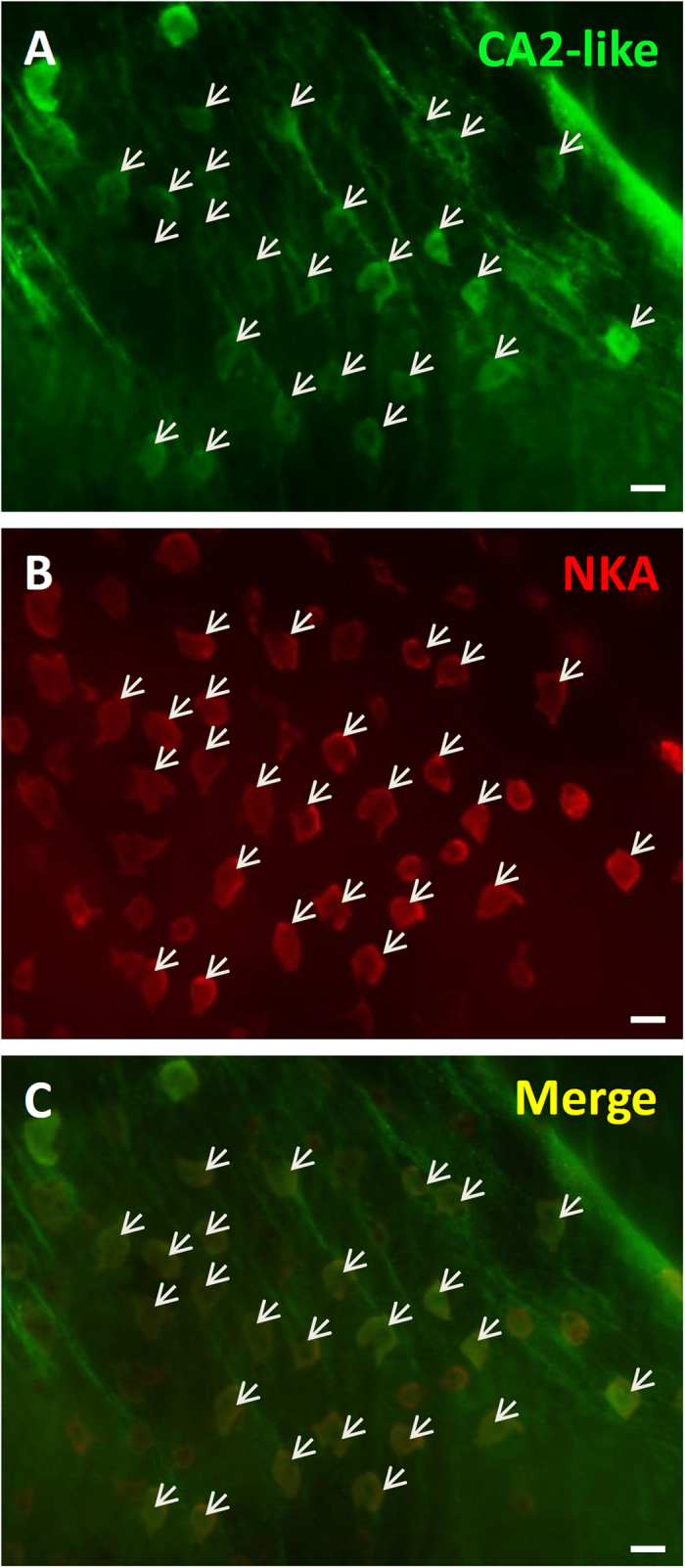
Localization of CA2-like proteins in ionocytes of larval skin. Immunohistochemistry was used to label medaka CA2-like (**A,C**). Na^+^/K^+^-ATPase (NKA) immunocytochemistry (**B,C**) was used to label ionocytes. CA2-like was co-expressed with NKA (arrows). Scale bars: 10 μm.

In addition to transcripts, CA2-like (Fig. 5A–C) protein was also colocalized with NKA in yolk sac ionocytes. Western blot analysis was used to verify the specificity CA2-like antibodies (supplementary Fig. S2). A single band of CA2-like was found at approximately 30 kDa. No band was shown in the negative control (omission of the primary antibody).

### The expression levels of NHE, CA, AE, and NKCC in the gills of medaka correlate with the salinity or pH of the SW to which they are acclimated

**Figure 6:**
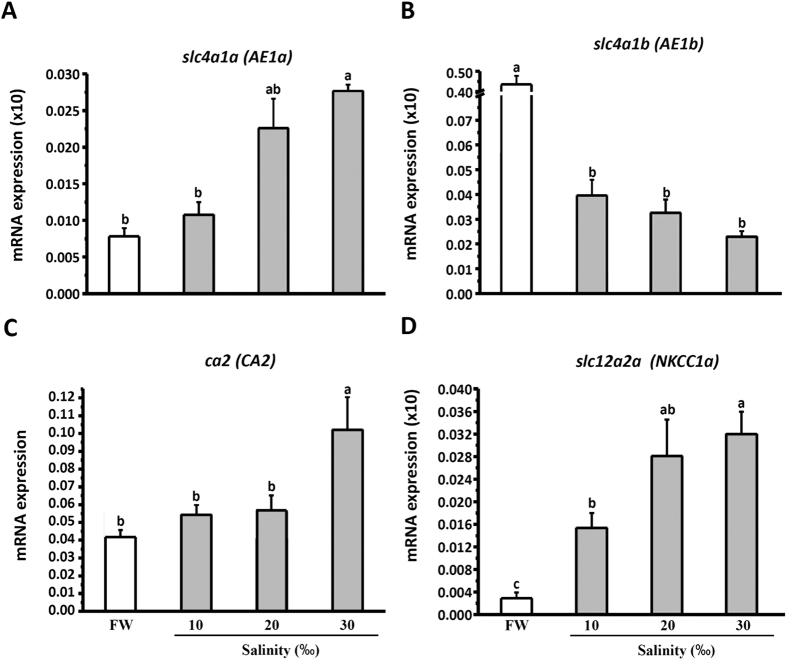
The expression of mRNA transcribed from *slc4a1a* (**A**), *slc4a1b* (**B**), *ca2-like* (**C**), and *slc12a2a* (**D**) in gill of adult medaka acclimated to FW or 10‰, 20‰, or 30‰ SW for 1 week. Data are presented as the means ± SEM (*n* = 4–5). Different letters indicate a significant difference (one-way ANOVA, Tukey’s comparison, *p* < 0.05). All results were normalized to *rpl7*.

Real-time qPCR was used to examine transcript levels of *slc4a1a* (AE1a), *slc4a1b* (AE1b), *ca2-like* (CA2-like), and *slc12a2a* (NKCC1a) in gills of adult medaka acclimated to FW or 10‰, 20‰, or 30‰ SW for 1 week. We observed that the levels of *slc4a1a*, *ca2-like*, and *slc12a2a* increased with salinity, and significant differences were identified between the FW and 30‰ SW groups (Fig. 6A,C,D). In contrast, *slc4a1b* level decreased with salinity; the FW and 30‰ SW groups also exhibited significant differences in *slc4a1b* expression (Fig. 6B).

**Figure 7:**
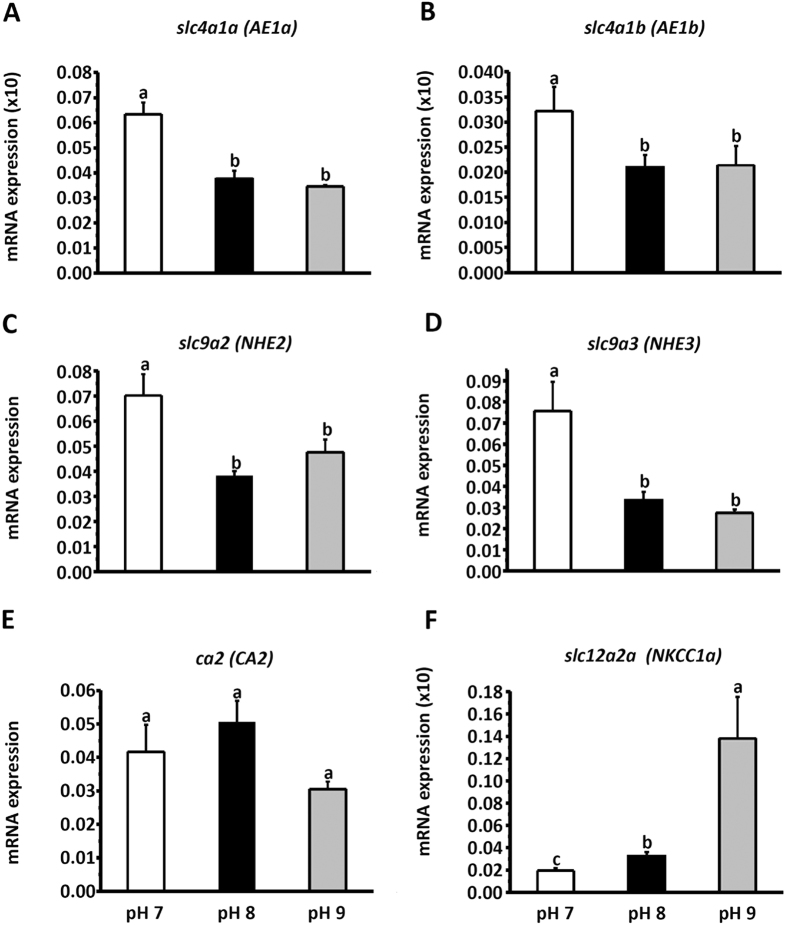
The expression of mRNA transcribed from *slc4a1a* (**A**), *slc4a1b* (**B**), *slc9a2* (**C**), *slc9a3* (**D**), *ca2-like* (**E**), and *slc12a2a* (**F**) in gills of adult medaka acclimated to acidified SW (pH 7), normal SW (pH 8), or alkaline SW (pH 9) for 1 week. Data are presented as the means ± SEM (*n* = *5*). Different letters indicate a significant difference (one-way ANOVA, Tukey’s comparison, *p* < 0.05). All results were normalized to *rpl7*.

We also acclimated medaka to SW with different pH (pH 7, pH 8, or pH 9) to examine gene expression in the gills of fish under pH challenge. After acclimation to acidified SW (pH 7) for 1 week, mRNA levels of *slc4a1a*, *slc4a1b*, *slc9a2* (NHE2), and *slc9a3* (NHE3) were significantly increased (Fig. 7A–D), whereas the mRNA level of *slc12a2a* was slightly decreased (Fig. 7F). In contrast, the level of *slc12a2a* was increased 7-fold after acclimating to pH 9-SW (Fig. 7F), whereas the expression of other genes was unaffected under the same conditions. The expression of *ca2-like* was not significantly different among fish acclimated to different pH (Fig. 7E).

## Discussion

Here, SIET was applied to measure H^+^ and Cl^−^ secretion by the skin ionocytes of medaka larva acclimated to SW. Our previous study^13^ and the present study (Fig. 1) revealed that H^+^ secretion of SW-type ionocytes was significantly suppressed by EIPA, DIDS, and AZ, suggesting that NHE, AE, and CA are involved in H^+^ secretion by skin ionocytes. More importantly, these inhibitors also significantly suppressed Cl^−^ secretion of ionocytes (Fig. 2). Exposing larvae to 1% CO_2_ hypercapnic SW stimulated not only H^+^ secretion, but also Cl^−^ secretion (Fig. 3) by ionocytes. These results show that H^+^ secretion is somehow linked to Cl^−^ secretion by SW-type ionocytes. NHE2 and NHE3 were previously identified in the SW-type ionocytes of medaka, where they are suggested to play a critical role in H^+^ secretion^13^,^24^. In this study, AE1 and CA2-like were further identified in SW-type ionocytes (Figs 4 and 5). These protein isoforms (NHE2/3, AE1, and CA2-like) are suggested to be the targets blocked by the inhibitors used in this study.

The CA2-like protein is localized to branchial ionocytes in several SW fishes, including killifish (*Fundulus heteroclltus*)^25^, flounder (*Platichthys flesus*)^16^, and mudskipper (*Periophthalmodon schlosseri*)^17^. The protein/mRNA level or enzyme activity of branchial CA has been analyzed in euryhaline species subjected to salinity changes. In killifish (*Fundulus heteroclltus*), the mRNA level of CA was higher in SW than in FW^26^. However, no difference in protein level was found between FW- and SW-acclimated individuals of flounder (*Platichthys flesus*)^16^,^27^ or pufferfish (*Tetraodon nigroviridis*)^18^. Branchial CA activity increased with salinity in salmon (*Oncorhynchus kisutch*)^28^ and tilapia (*Oreochromis mossambicus*)^29^, whereas it was unchanged by salinity in European eels (*Anguilla anguilla*)^30^. In medaka, we found that branchial CA2-like mRNA levels increased with salinity (Fig. 6), which is consistent with the findings in killifish, salmon, and tilapia. The upregulation of CA in gills might reflect the need for acid-base regulation in fish. It was reported that fish face acidosis and low blood HCO_3_^−^ levels after SW transfer^31^,^32^,^33^, and suggested that upregulation of branchial CA enhances CO_2_ hydration and subsequently HCO_3_^−^ reclamation via fish gills^34^,^35^,^36^. Toadfish (*Opsanus beta*) subjected to salinity challenges exhibit elevated acid secretion by the gills and kidneys to compensate for the loss of base (HCO_3_^−^) from intestine^36^,^37^. The finding (Fig. 1) that blocking CA with AZ suppressed H^+^ secretion by ionocytes demonstrates that CA is indeed required for acid secretion by SW fish. However, a possible physiological linkage between increased CA expression or activity and salt secretion mechanisms cannot be excluded. Such a linkage was hinted by the findings that the CA inhibitor suppressed Cl^−^ secretion, and that CA2-like mRNA expression was unaffected by different pH conditions (Figs 2B and 7E, also see below).

In zebrafish, AE1b was found to be the dominant isoform of AE1 in the gills, and localized to the basolateral membrane of ionocytes^19^. In medaka, AE1b mRNA was also found in the skin ionocytes of larvae acclimated to FW^24^. In euryhaline fishes, including pufferfish (*Tetraodon nigroviridis*) and milkfish (*Chanos chanos*), AE1 was found to be localized to the basolateral membrane of ionocytes in both FW- and SW-acclimated individuals, as determined using an antibody against tilapia AE1^18^,^20^. Furthermore, we found that both AE1a and AE1b mRNA are expressed in most SW-type ionocytes in medaka larvae (Fig. 4). Previously, little was known about the role of AE1 in the ionocytes of SW fish. In this study, H^+^ secretion of ionocytes was reduced by DIDS exposure for 30 min (Fig. 1). In addition, mRNA levels of both AE1a and AE1b were upregulated by acidified SW (pH 7) (Fig. 7). These results suggest that basolateral AE1 is also involved in acid secretion by SW-type ionocytes. In an early study, acid secretion by whole animals was found to be stimulated by DIDS treatment in sculpin (*Myoxocephalus octodecimspinosus*)^38^. The result from sculpin might represent systematic and long-term effects of DIDS on acid-base regulation of animals, because the animals were exposed to DIDS for 3 h. The real-time qPCR data showed that AE1a was upregulated, while AE1b was downregulated, in the gills of medaka after SW acclimation (Fig. 6A,B), implying that AE1a might be more important than AE1b for SW acclimation. However, it should be noted that the expression level of AE1b was comparable to that of AE1a in SW-acclimated medaka. Therefore, we concluded that both isoforms are required for SW-type ionocytes. More importantly, this study shows for the first time that AE1 in ionocytes of SW fish is involved in acid and salt secretion.

**Figure 8:**
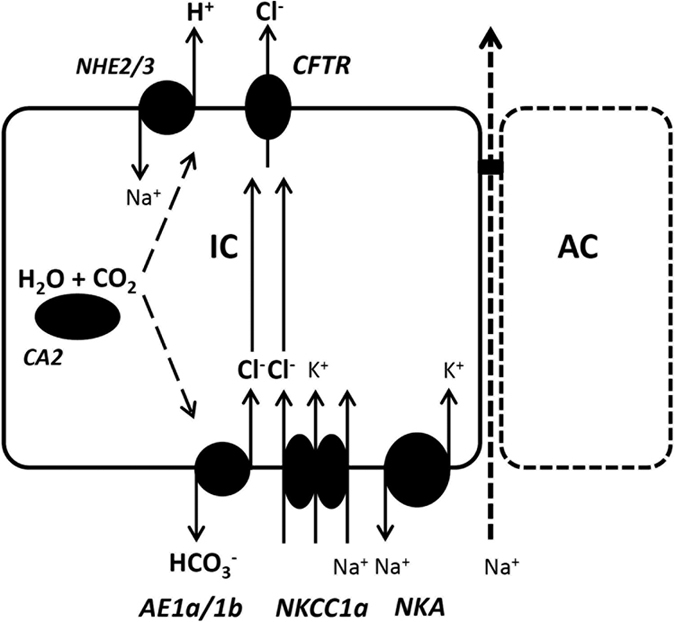
Proposed model of H^+^ and Cl^−^ secretion by ionocytes in SW medaka. Refer to discussion for details.

Our previous study suggested that NHE2 and NHE3 play critical roles in the acid secretion of SW-type ionocytes by transporting H^+^ into SW^13^. In this study, we further observed that CA2-like and AE1a/1b are involved in acid secretion by ionocytes. In addition, basolateral localization of AE1 was reported in pufferfish (*Tetraodon nigroviridis*) and milkfish (*Chanos chanos*)^18^,^20^. Taken together, we propose a model for the mechanism of acid secretion by SW-type ionocytes (Fig. 8). In this model, CA2-like catalyzes CO_2_ hydration and produces intracellular H^+^ and HCO_3_^−^, and H^+^ and HCO_3_^−^ are then transported out of the ionocytes via apical NHE2/3 and basolateral AE1a/1b, respectively. This model is similar to that proposed for acid-secreting ionocytes (HR cells) of zebrafish^5^,^19^.

In this study, treatment with DIDS, AZ, or EIPA was also found to suppress Cl^−^ secretion by ionocytes (Fig. 2). A similar observation was reported in an early *in vitro* study: basolateral treatments of DIDS and amiloride (an analogue of EIPA) were found to suppress short-circuit currents (Cl^−^ secretion current) of opercular membrane isolated from killifish (*Fundulus heteroclitus*)^39^. In this study, DIDS treatment suppressed 35% of H^+^ secretion and 57% of Cl^−^ secretion, suggesting that AE1-mediated HCO_3_^−^/Cl^−^ exchange not only contributes to acid-base regulation, but also to Cl^−^ secretion of SW-type ionocytes. Basolateral NKCC1 is well known to carry Cl^−^ from interstitial fluid into ionocytes for Cl^−^ secretion^5^,^24^. Here, we suggest that basolateral AE1 is another transporter that can carry Cl^−^ into the ionocytes of SW fish; in other words, AE1 plays a dual role, affecting both acid-base regulation and Cl^−^ secretion of ionocytes, accounting for the linkage of these two functions. If this is the case, how do apical NHE and cytosolic CA influence Cl^−^ secretion? As depicted in our model, NHE2/3, CA2-like, and AE1 function together to enable H^+^ secretion and bicarbonate reclamation, and inhibiting any one of these three proteins can suppress the entire process, thereby hindering H^+^ and Cl^−^ secretion.

In this study, we further compared the contribution of NKCC1 and AE1 to Cl^−^ secretion by blocking NKCC1 and/or AE1 with inhibitors. We found that Cl^−^ secretion by ionocytes was reduced 63% by bumetanide, 47% by DIDS, and 81% by combined treatment with both inhibitors (Fig. 2D), suggesting that both transporters are involved in Cl^−^ secretion, and the contribution of NKCC1 is greater than that of AE1. In this study, larvae were not killed by short-term treatments (10 min), but were killed by long-term treatment with dual inhibitors, suggesting that complete blockade of Cl^−^ secretion is lethal. The branchial mRNA levels of AE1a and AE1b were elevated in acidified SW (pH 7); in contrast, NKCC1a was elevated in pH 9-SW (Fig. 7). These results suggest that AE1-mediated Cl^−^ secretion may be important for fish with acidosis; on the other hand, NKCC1-mediated Cl^−^ secretion may be essential for fish with alkalosis.

Basolateral NKCC1-mediated Cl^−^ secretion has been observed in variety of salt-secreting epithelia in animals^40^,^41^,^42^. It is generally accepted that electro-neutral NKCC1 is driven by a Na^+^ gradient established by a Na^+^ pump to transport Cl^−^ against the high intracellular Cl^−^ gradient. However, little is known about basolateral AE-mediated Cl^−^ secretion in animals. Basolateral AE2 in a unique and powerful HCl-secreting cell, the parietal cell of the gastric gland in mammal, was found to play a critical role in transporting Cl^−^ into cells by exchange with intracellular bicarbonate generated by cytosolic CA^43^,^44^,^45^. In addition, basolateral NHE4 was shown to be coupled with AE2 to facilitate extracellular removal of HCO_3_^−^46. In pufferfish, CA2-like was demonstrated to physically associate with basolateral AE1 in ionocytes^18^. It is possible that CA2-like, AE1, and a putative NHE in the basolateral membrane of ionocytes may function together as a protein metabolon to facilitate Cl^−^/HCO_3_^−^ exchange. This assumption needs to be investigated in the future.

In FW-acclimated medaka and zebrafish, acid secretion by ionocytes was demonstrated to be associated with ammonia excretion and Na^+^ uptake^23^,^47^. In SW-acclimated medaka, our previous study showed that acid secretion can facilitate ammonia excretion^13^. In this study, acid secretion was shown to be linked with Cl^−^ secretion in SW. Taken together, these findings reveal that acid-base regulation, ammonia excretion, and osmoregulation of teleosts are tightly linked in ionocytes of both FW- and SW-acclimated fish.

## Materials and Methods

### Experimental animals

Mature Japanese medaka (*Oryzias latipes*) were reared in circulating tap water at 28 °C with a 14:10 h light-dark photoperiod. The females spawned every day, and fertilized egg clusters were collected from the belly of a female. The egg clusters were placed in a fish net, rinsed gently with running tap water and crumpled by fingers from outside the net. This treatment facilitates separation of collected egg clusters into single eggs^48^.

Medaka embryos usually hatched at 7–8 days postfertilization (dpf), and newly hatched larvae were used for the experiments. During the experiments, larvae were not fed, and the media were changed daily to maintain water quality. The experimental protocols were approved (no. 95013) by the National Taiwan Normal University Animal Care and Utilization Committee, and were carried out in accordance with the approved guidelines.

### Acclimation experiments

#### Embryo

SW was prepared by adding the appropriate amounts of sea salt (Instant Ocean, Aquarium System, Mentor, OH) to redistilled water (dH_2_O). Normal fresh water (NW) contained (in mM) 0.5 NaCl, 0.2 CaSO_4_, 0.2 MgSO_4_, 0.16 KH_2_PO_4_, and 0.16 K_2_HPO_4_ (pH 6.8–7.0, adjusted with NaOH and HCl). For SW acclimation, fertilized eggs were transferred to NW for the first 2 days and then directly transferred to 30‰ SW (pH 8.0, adjusted with NaOH and HCl) for 6 days. For short-term CO_2_ exposure, medaka larvae were directly transferred to hypercapnic SW (1% CO_2_; pH 7.4) for 10 min before SIET measurement. For long-term CO_2_ exposure, the embryos were transferred to hypercapnic SW (1% CO_2_; pH 7.4) for 6 days.

#### Adult fish

For salinity acclimation, adult medaka were transferred to 10‰ SW for 2 days and then transferred to 10, 20 or 30‰ SW for 1 week. Medaka of control group (FW group) were transferred to another FW tanks at the same time. For pH acclimation, fish were transferred to 10‰ SW for 2 days and then transferred to 30‰ SW with pH 7, pH 8, or pH 9 (adjusted with NaOH and HCl) for 1 week. In these experiments, 3 individuals were kept in 1 tank, each group consisted of 4–5 tanks (12–15 individuals).

### Scanning ion-selective electrode technique (SIET)

The SIET was used to measure H^+^ and Cl^−^ activities at ionocyte surface of larvae. Glass capillary tubes (no. TW 150-4, World Precision Instruments, Sarasota, FL, USA) were pulled on a Sutter P-97 Flaming Brown pipette puller (Sutter Instruments, San Rafael, CA, USA) into micropipettes with tip diameters of 3–4 μm. These were then baked at 120 °C overnight and coated with dimethyl chlorosilane (Sigma-Aldrich) for 30 min. The micropipettes were backfilled with a 1-cm column of electrolytes and frontloaded with a 20–30 μm column of liquid ion-exchange cocktail (Sigma-Aldrich) to create an ion-selective microelectrode (probe). The following ionophore cocktails (and electrolytes) were used: H^+^ ionophore I cocktail B (40 mM KH_2_PO_4_ and 15 mM K_2_HPO_4_) and Cl^−^ ionophore I cocktail B (100 mM NaCl). The details of the system were described in previous reports^13^,^21^. To calibrate the ion-selective probe, the Nernstian property of microelectrode was measured by placing the microelectrode in a series of standard solutions (pH 7, 8, and 9 for the H^+^ probe; 10, 100 and 1000 mM NaCl for Cl^−^ probe)^49^. By plotting the voltage output of the probe against log[H^+^] values, a linear regression yielded a Nernstian slope of 58.6 ± 0.8 (*n* = 10)^13^ for H^+^ and −59.3 ± 3.4 (*n* = 10)^21^ for Cl^−^. The SIET was performed at room temperature (26–28 °C) in a small plastic recording chamber (radius: 2 cm; height: 0.5 cm) filled with 2 ml of recording medium. The recording medium of SW contained 350.9 mM NaCl, 45.7 mM MgCl_2_·6H_2_O, 24.2 mM Na_2_SO_4_, 8.9 mM CaCl_2_·2H_2_O, 7.8 mM KCl, 2 mM NaHCO_3_, 300 μM Tricine and 0.3 mg l^−1^ ms222. pH values of SW recording media was adjusted to 8.0. Before the measurement, an anesthetized larva was positioned in the center of the chamber with its lateral side contacting the base of the chamber.

### Measurement of H^+^ gradient and Cl^−^ flux at specific cells

To record the surface H^+^ gradient (Δ[H^+^]) and Cl^−^ flux at specific cells, the probe was moved to a position 1–2 μm above the apical membrane of cells. The voltage difference in microvolts was measured by probing orthogonally to the surface at 10-μm intervals. Ten replicates of recordings at an ionocyte or keratinocyte were performed, and the median value was used for calculating the Δ[H^+^] or Cl^−^ flux of cell. Voltage differences obtained were converted into ionic gradients or flux by ASET software following previous reports^13^,^21^. Voltage differences obtained from ASET software were converted to a concentration gradient using the following equation: Δ*C* = *C*_b_ × 10(Δ*V*/*S*) − *C*_b_, where Δ*C* is the concentration gradient between two points, *C*_b_ is the background ion concentration, Δ*V* is the voltage gradient obtained from ASET software, and *S* is the Nernst slope of the electrode. The concentration gradient was subsequently converted to ionic flux using Fick’s law of diffusion: *J* = *D* (Δ*C*)/Δ*X*, where *J* (pmol·cm^−2^·s^−1^) is the net flux of the ion, *D* is the diffusion coefficient of the ion (in SW: 8.75 × 10^−5^ cm^2^/s for H^+^ and 1.77 × 10^−5^ cm^2^/s for Cl^−^)^50^, ΔC (pmol/cm^−3^) is the concentration gradient, and Δ*X* (cm) is the distance between the two points. We usually recorded 2–3 ionocytes in the same larva, and 1 larva was recorded for no longer than 20 min.

### Addition of DIDS, AZ, EIPA, and Bumetanide

4, 4′-diisothiocyanatostilbene-2, 2′-disulphonic acid (DIDS; 400 μM), acetazolamide (AZ; 200 μM), 5-(N-ethyl-N-isopropyl)-amiloride (EIPA; 400 μM), and bumetanide (Bumex; 400 μM) were obtained from Sigma-Aldrich, and were used to respectively suppress AE, CA, NHE, and NKCC in this study. Stock solutions of DIDS, AZ, and EIPA were prepared by dissolving them in DMSO (Sigma-Aldrich). Stock solutions of bumetanide were prepared by dissolving it in ethanol (Sigma-Aldrich). The final concentration of DMSO or ethanol in the working solutions (including the control group) was 0.1%. 0.1% DMSO was added to the control group. A preliminary test (supplementary Fig. S3) showed that ethanol did not affect Cl^−^ secretion by ionocytes.

Before SIET recording, larvae were incubated in 1 ml of SW medium with DIDS, AZ, EIPA, or bumetanide (pH 8.0, adjusted with NaOH and HCl) for 30 min. After incubation, the larvae were transferred to SW recording medium that did not contain any inhibitors.

### Preparation of RNA

Adult medaka were anesthetized on ice, killed by spinal transection, and the gills were excised and immediately transferred to PBS (0.09% NaCl in 0.1 M phosphate buffer). Gills (40–50 mg) collected from 3 individuals in the same tank were pooled as 1 sample. Samples were homogenized in 0.5 ml Trizol Reagent (Invitrogen, Carlsbad, CA, USA), and DNA contamination was removed with DNase I (Promega, Madison, WI, USA). Total RNA was purified using a MasterPure™ RNA Purification Kit (Epicentre Biotechnologies, Madison, WI, USA). The amount of total RNA was determined using a NanoDrop 1000 spectrophotometer (Thermo Scientific, Wilmington, DE, USA). All RNA pellets were stored at −20 °C for less than 1 week.

### Reverse-transcription polymerase chain reaction (RT-PCR) analysis

For complementary (c)DNA synthesis, 5 μg of total RNA was reverse-transcribed in a final volume of 20 μl containing 0.5 mM dNTPs, 2.5 μM oligo(dT)_20_, 250 ng of random primers, 5 mM dithiothreitol, 40 units of an RNase inhibitor, and 200 units of SuperScript III RT (Invitrogen, Carlsbad, CA, USA) for 1 hr at 55 °C, followed by incubation at 70 °C for 15 min.

### Quantitative real-time polymerase chain reaction (qRT-PCR) analysis

After (c)DNA synthesis, mRNA expression of the target gene was measured by qRT-PCR with the Roche LightCycler 480 System (Roche Applied Science, Mannheim, Germany). Specific primers for all genes were designed using Primer Premier software (version 6.0; PREMIER Biosoft International, Palo Alto, CA, USA). The accession numbers and primer sequences of target genes were showed in supplementary Table S1. PCRs contained 3.2 ng of cDNA, 50 nM of each primer, and LightCycler^®^ 480 SYBR Green I Master (Roche, Mannheim, Germany) in a final volume of 10 μl. All qRT-PCRs were performed as follows: 1 cycle of 50 °C for 2 min and 95 °C for 10 min, followed by 40 cycles of 95 °C for 15 s and 60 °C for 1 min (the standard annealing temperature of all primers). PCR products were subjected to a melting-curve analysis, and representative samples were electrophoresed to verify that only a single product was present. A no-template control reaction was conducted with sterile water to determine the levels of background. In addition, a no-reverse transcriptase control was used to check for genomic DNA contamination. The standard curve of each gene was checked in a linear range with ribosomal protein L7 (*rpl7*) as an internal control^13^,^24^. The mRNA expression of target gene in each sample was normalized to its reference gene (*rpl7*). The calculation of relative mRNA levels was based on the comparative Ct method^51^.

### RNA probe synthesis

A fragment of AE1a (*slc4a1a)* and AE1b (*slc4a1b)* were obtained by PCR and inserted separately into the pGEM-T Easy vector. The primer sequences of target genes were as follows: (*slc4a1a*: F-5′ GGAGTCTCAGATTACCACGCT 3′, R-5′ ATCATCTCCAGGTTCGTCGTCAAAT 3′; *slc4a1b*: F-5′ TGGATGTTTCTTTATTGCCTTTT 3′, R-5′ TCATTTGGATGGTATTTCTTTGG 3′). The inserted fragments were amplified with the T7 and SP6 primers by PCR, and the products were respectively used as templates for *in vitro* transcription with T7 and SP6 RNA polymerase (Roche, Penzberg, Germany). Digoxigenin (DIG)-labeled RNA probes were examined using RNA gels and dot blot assays to confirm their quality and concentrations.

### *In situ* hybridization

Medaka larvae were anesthetized on ice and then fixed with 4% paraformaldehyde in a phosphate-buffered saline (PBS) solution at 4 °C overnight. Afterwards, samples were washed with diethylpyrocarbonate (DEPC)-PBST (PBS with 0.1% Tween-20) three times (10 min/wash). After a brief rinse with PBST, embryos were refixed with 4% paraformaldehyde for another 20 min. After PBST washing, samples were incubated with hybridization buffer (HyB: 50% formamide, 5× SSC (saline-sodium citrate), and 0.1% Tween 20) at 65 °C for 5 min, and then with HyB containing 500 μg/ml yeast tRNA at 65 °C for 8 h before hybridization. After overnight hybridization with 1000 ng/ml DIG-labeled antisense or sense RNA probes, embryos were serially washed with 50% formamide-2× SSC (at 65 °C for 20 min), 2× SSC (at 65 °C for 10 min), 0.2× SSC (at 65 °C for 30 min, twice), and PBST at room temperature for 10 min. Afterwards, embryos were reacted with an alkaline phosphatase-coupled anti-DIG antibody (diluted 1: 10000) and then treated with nitro blue tetrazolium (NBT) and 5-bromo-4-chloro-3-indolyl phosphate (BCIP) for the alkaline phosphatase reaction. For double staining, *in situ*-hybridized samples were subjected to immunocytochemistry.

### Antibodies

The primary antibodies used in this study include: (1) the CA2-like antibody: a rabbit polyclonal antibody against the CA2a of zebrafish^52^ (kindly provided by Dr. S. Hirose in Department of Biological Sciences, Tokyo Institute of Technology); (2) the Na^+^-K^+^-ATPase (NKA) antibody: a monoclonal antibody against the α-subunit of the avian NKA (Developmental Studies Hybridoma Bank, University of Iowa, Ames, IA).

### Immunohistochemistry (IHC)

The protocol for CA2-like IHC was modified from a previous report^53^. The embryos were fixed with 4% paraformaldehyde (PFA) at 4 °C overnight. Afterwards, medaka embryos were washed with 1x PTw (1x PBS at pH 7.3, 0.1% Tween) for 3 × 5 min, and then stored in 100% MeOH at −20 °C for at least two days. Fixed embryos were rehydrated with 1x PTw for 3 × 10 min, and then embryos were incubated in 150 mM Tris-HCl at pH 9.0 for 5 min, followed by heating at 70 °C for 15 min. After heating treatment, embryos were incubated in 1x PTw for 2 × 10 min and then washed in dH_2_O for 2 × 5 min. Subsequently, all embryos were treated with acetone for 20 min at −20 °C. After acetone treatment, the embryos were sequentially washed in dH_2_O for 2 × 5 min and 1x PTw for 2 × 5 min, and then incubated in blocking buffer (B-buffer) (10% sheep serum, 0.8% Triton X-100, 1% BSA in 1x PTw) for 3 h at 4 °C. After blocking, the embryos were incubated with the CA2-like antibody (diluted 1: 300) in the incubation buffer (I-buffer) (1% sheep serum, 0.8% Triton X-100, 1% BSA in 1x PTw) at 4 °C for three days. To remove residual primary antibody, embryos were sequentially washed with PBS-TS (10% sheep serum, 1% Triton X-100 in 1x PBS) for 3 × 1 h, with PBS-T (1% Triton X-100 in 1x PBS) for 2 × 10 min, and again with PBS-TS for 2 × 1 h. The embryos were incubated with goat anti-rabbit IgG conjugated with Alexa Fluor 488 (Molecular Probes, Carlsbad, CA) (1:500 dilution) in the dark for two and half days, and then washed with PBS-TS for 3 × 1 h and with 1x PTw for 2 × 1 h. For NKA staining, the protocol was conducted as described by previous reports^13^,^23^. Finally, images were obtained using a fluorescence microscope (Axioplan 2 Imaging, Carl Zeiss Oberkochen, Germany).

### Statistical analysis

Data are expressed as the mean ± SEM (*n*, number of larvae or ionocytes). Values from each condition were analyzed using one-way analysis of variance (ANOVA) followed by Tukey’s pairwise comparisons. Student’s unpaired *t*-test (two-tailed) was used for simple comparisons of two means. Significance was set at *α* level of 0.05.

## Additional Information

**How to cite this article**: Liu, S.-T. *et al*. Salt secretion is linked to acid-base regulation of ionocytes in seawater-acclimated medaka: new insights into the salt-secreting mechanism. *Sci. Rep.* **6**, 31433; doi: 10.1038/srep31433 (2016).

## Supplementary Material

Supplementary Information
