# Supplementary material for: Salt secretion is linked to acid-base regulation of ionocytes in seawater-acclimated medaka: new insights into the salt-secreting mechanism
Source: Sci Rep. 2016 Aug 11;6:31433. doi: 10.1038/srep31433 (PMC4980601; doi:10.1038/srep31433)

**Supplementary information**

**Salt secretion is linked to acid-base regulation of ionocytes in seawater-acclimated medaka: new insights into the salt-secreting mechanism**

Sian-Tai Liu^1^, Jiun-Lin Horng^2^, Po-Yen Chen^1^, Pung-Pung Hwang^3^*, Li-Yih Lin^1^*

^1^ Department of Life Science, National Taiwan Normal University, Taipei, Taiwan

^2^ Department of Anatomy and Cell Biology, Taipei Medical University, Taipei, Taiwan

^3^ Institute of Cellular and Organismic Biology, Academia Sinica, Taipei, Taiwan

* These two authors contributed equally to this study.

Correspondence to Dr. L. Y. Lin, Department of Life Science, National Taiwan Normal University, Taipei 116, Taiwan

Tel: +8862-77346316; Fax: +8862-29312904; E-mail: linly@ntnu.edu.tw

**Materials and methods**

***Western Blot******s***

Gills were washed twice with cold PBS, and sampled in homogenization buffer (100 mM imidazole, 5 mM EDTA, 200 mM sucrose, and 0.1% sodium deoxycholate; pH 7.6), and then centrifuged at 4°C and 10,000 rpm for 10 min. The supernatant (a volume equivalent to 40 μg protein) was supplemented with electrophoresis sample buffer (250 mM Tris-base, 2 mM Na_2_EDTA, 2% SDS, and 5% dithiothreitol), and then incubated at 95°C for 10 min. The denatured samples were subjected to 10% sodium dodecyl sulfate (SDS)-polyacrylamide gel electrophoresis then transferred to polyvinylidene difluoride membranes (Millipore, Billerica, CA, USA). After blocking in 5% nonfat milk, the blots were incubated with a rabbit anti-tilapia AE1 polyclonal antibody (diluted 1:500), and a rabbit anti-zebrafish CA2a polyclonal antibody (diluted 1:1000), respectively at 4℃ overnight. Samples were washed with PBST and incubated with a horseradish peroxidase-conjugated goat anti-rabbit IgG antibody (dilute 1:5,000; Millipore) at room temperature for another 2 h. Blots were visualized with an enhanced chemiluminescence system (Millipore). The image was captured using an ImageQuant 4000 system (GE Healthcare, Buckinghamshire, UK).

**Supplementary Table S1.** Primer sequences for quantitative real-time PCR analysis. For the primers designed in our lab, intron/exon boundary-spanning primers were preference to minimize the genomic DNA contamination. Primer efficiency values are ranging from 90% to 110%.

| Name (accession number) | Primer Sequence | Amplicon lengths |
| --- | --- | --- |
| *slc4a1a* | F 5’ TTGAATGAGCTCCAAGGCAACACC 3’ | 189 bp |
| (ENSORLG00000002557) | R 5’ CAGATTGCTGGCGTTCATGTCCAA 3’ |  |
| *slc4a1b* | F 5’ TGGCCAACAAGAAGGAGATCAGGT 3’ | 199 bp |
| (ENSORLG00000005495) | R 5’ AGGAAGTCCATGGCTCCCACTAAA 3’ |  |
| *slc9a2* | F 5’ ATCGTCTGTTGTGCCCTC 3’ | 167 bp |
| (ENSORLG00000012399) | R 5’ CAGTTCCACTCGTGCTCT 3’ |  |
| *slc9a3* | F 5’ ATGCCTGATGTCACTGCT 3’ | 186 bp |
| (ENSORLG00000009128) | R 5’ GTGTCGGTGCTGCTTCCT 3’ |  |
| *ca2-like* | F 5’ ACGGACATTCCATCCAAGTGACCT 3’ | 155 bp |
| (ENSORLG00000012745) | R 5’ TTTGGTTCCGTTCACCGTGTGTTC 3’ |  |
| *slc12a2a* | F 5’ TCTGGTGGCTGTTTGATGATG 3’ | 446 bp |
| (ENSORLG00000019274) | R 5’ AGGCAGGCTTATGACGATGA 3’ |  |
| *rpl7* | F 5’ GAGATCCGCCTGGCTCGTA 3’ | 107 bp |
| (ENSORLG00000007967) | R 5’ GGGCTGACTCCGTTGATACCT 3’ |  |

**Supplementary Figure S1.** Negative controls of *in situ* hybridization of *slc4a1a* and *slc4a1b* in medaka larvae. Sense probes were used as negative controls. Scale bar: 100 µm.


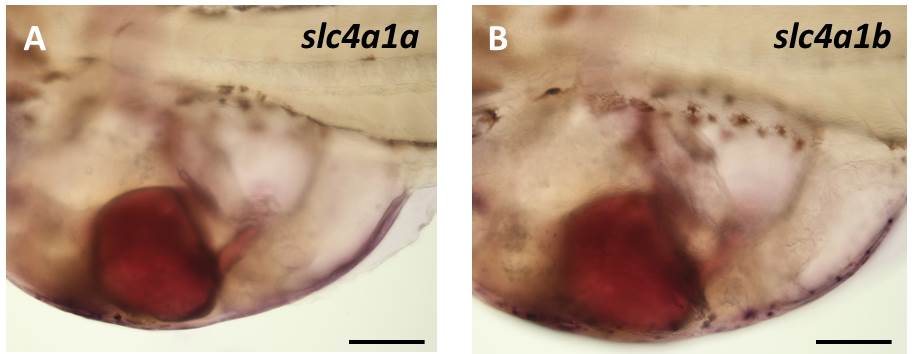


**Supplementary Figure S2.** Western blot analysis of CA2-like in gills of SW-acclimated medaka. Arrows indicate putative sizes of CA2-like (30 kDa). Positive (+) indicates the sample treated with primary antibody. Negative (-) indicates the sample without treatment of primary antibody.


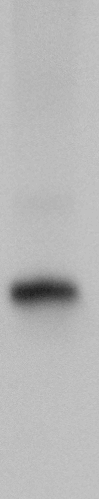

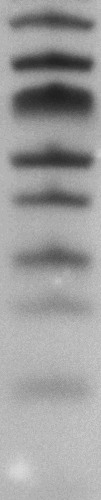

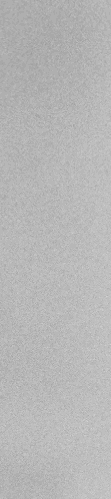


**100k**

**70k**

**55k**

**40k**

**35k**

**25k**

**15k**

**M**

**CA2**

**-**

**like**

**+**

**CA2**

**-**

**like**

**-**

**30k**

**Supplementary Figure S3.** The effect of 0.1% ethanol on Cl^-^ flux of ionocytes in SW-acclimated medaka larvae. Data are presented as the means ± SEM (two-tailed unpaired Student’s *t*-test). The numbers of analyzed cells (from 6-7 larvae) are shown in parentheses.


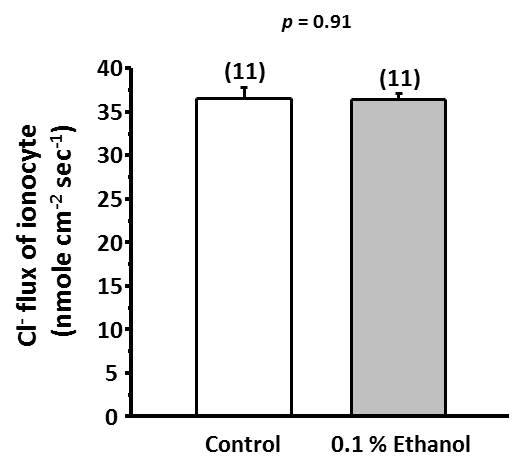

Supplement: Supplementary Information [file srep31433-s1.docx]
